# Contextual Interference in Complex Bimanual Skill Learning Leads to Better Skill Persistence

**DOI:** 10.1371/journal.pone.0100906

**Published:** 2014-06-24

**Authors:** Lisa Pauwels, Stephan P. Swinnen, Iseult A. M. Beets

**Affiliations:** 1 KU Leuven, Movement Control and Neuroplasticity Research Group, Department of Kinesiology, Group Biomedical Sciences, Leuven, Belgium; 2 KU Leuven, Leuven Research Institute for Neuroscience & Disease (LIND), Leuven, Belgium; Harvard Medical School, United States of America

## Abstract

The contextual interference (CI) effect is a robust phenomenon in the (motor) skill learning literature. However, CI has yielded mixed results in complex task learning. The current study addressed whether the CI effect is generalizable to bimanual skill learning, with a focus on the temporal evolution of memory processes. In contrast to previous studies, an extensive training schedule, distributed across multiple days of practice, was provided. Participants practiced three frequency ratios across three practice days following either a blocked or random practice schedule. During the acquisition phase, better overall performance for the blocked practice group was observed, but this difference diminished as practice progressed. At immediate and delayed retention, the random practice group outperformed the blocked practice group, except for the most difficult frequency ratio. Our main finding is that the random practice group showed superior performance persistence over a one week time interval in all three frequency ratios compared to the blocked practice group. This study contributes to our understanding of learning, consolidation and memory of complex motor skills, which helps optimizing training protocols in future studies and rehabilitation settings.

## Introduction

In the field of practice organization for motor skill learning, contextual interference (CI) has been one of the most frequently discussed topics over the past three decades. The term, CI, was introduced by Battig [1] and refers to the interference that results from performing various tasks or skills within the context of practice. Shea and Morgan [2] were the first to establish this effect in motor skill learning. Since then, numerous investigations led to the finding that introducing high amounts of CI, by means of presenting multiple task variants in a randomized order, leads to inferior performance during acquisition but benefits learning, reflected by better retention and transfer tests, in contrast to a blocked practice schedule [3], [4]. This phenomenon denotes a distinction between performance and learning and is often referred to as the paradoxical opposing effects of CI during acquisition and retention.

The CI effect is quite a robust phenomenon in laboratory experiments using multi-segment movement tasks, coincident anticipation timing tasks, aiming tasks, movement patterning tasks and tracking tasks [4], [5]. Several theoretical explanations underlying the CI effect have been proposed of which the elaboration hypothesis [2], [6] and the action-plan reconstruction hypothesis [7], [8] are the most prominent ones. The elaboration hypothesis [2] states that the benefits of high CI are due to more elaborative and distinctive processing because multiple tasks reside together in working memory, whereas when practicing under low CI, only one task is present in working memory. However, Lee and Magill [7] proposed the action-plan reconstruction hypothesis stating that action plans will be forgotten because of alternating trials following a random practice schedule. Thus, high amounts of CI will result in more effortful reconstructive processing whereas the action plan in blocked practice will be remembered.

Nevertheless, these two hypotheses may have a common factor namely enhanced cognitive effort and processing when practicing under high CI and decreased cognitive activity and processing when CI is low [9]. Recently, Kantak and Winstein [10] suggested a novel point of view to clarify the distinction between performance and learning by proposing a motor behavior-memory framework that shows the evolution of motor memory processes. The behavior-memory framework highlights the importance of the temporal evolution of memory processes (i.e. encoding, consolidation and retrieval) and states that the efficiency of these processes can be reflected in performance measures at different time points. Such a framework is important because motor memory processes, for example encoding [11], [12] and consolidation [13], [14] processes, can be differentially affected by different practice structures.

Although numerous studies have confirmed the CI effect in rather simple tasks, the CI effect is much less explored in complex tasks that require more cognitive effort. Multiple studies have been carried out, supporting either of both hypotheses [4]. However, it remains to be seen which of the two is favorable. In addition, none of these accounts make distinct predictions with respect to the CI effect in more complex tasks [5]. As stated by Wulf and Shea [5], the key question is whether principles derived from simple task studies can be extended to complex skill learning, which is essential in real life. According to the aforementioned hypotheses, enhanced cognitive activity and processing through high CI are assumed to be critical for enhanced learning. But what if the nature of the task itself is more difficult requiring high attention and memory demands? Would high CI further benefit or rather perturb skill learning? This prompts questions about the boundary conditions of the CI effect. Previous studies examining the effect of CI in complex motor skills have led to contrary results [5]. While some studies succeeded in demonstrating clear benefits of high amounts of CI in complex skill learning [15]–[17], others did not find any evidence at all [18], [19]. Both the studies of Albaret and Thon [20] and Tsutsui et al. [21] yielded mixed results. Because these papers are related to our topic, a more in depth discussion about these papers can be found in the discussion section. Yet, Wulf and Shea [5] aimed to get an overall view of the CI effect in tasks of various difficulty levels and noted that random practice is generally effective when learning simple tasks, i.e. with low attention and memory demands, or when a person is experienced in a complex task, requiring reduced memory demands. In line with this notion, Shea et al. [22] proposed that the benefits of CI increased with increasing amounts of practice. This might suggest that a certain level of experience in a complex task is required in order to obtain benefit from randomized practice [22], [23].

Since many tasks in daily life require a good coordination between both hands, the purpose of this study was to explore the CI effect in a complex bimanual coordination task. However, defining “complexity” is a tough job [5]. Considering the distribution of brain activations involved in various bimanual tasks, we assume our task to be sufficiently complex as it requires higher cognitive functions [24]. In the current paper, three task variants were divided over multiple days of training. In accordance with both the elaboration and action-reconstruction hypotheses, we hypothesized that practicing under high (i.e. a randomized practice schedule) as compared to low CI will have detrimental effects during acquisition, but will benefit retention performance immediately and seven days after the last practice day. However, as discussed previously, Kantak and Winstein [10] highlighted the importance of the temporal evolution of memory processes. Thus, besides directly comparing retention performance between groups, which encompasses both encoding and consolidation processes, we also looked into processes occurring during the retention interval, i.e. post-acquisition. As one key feature of the CI effect is that temporary performance benefits should be sacrificed for long-term learning benefits [4], we hypothesized that practicing under a randomized practice schedule will result in better performance persistence across retention intervals. During these post-acquisition processes, a long-term memory representation will be generated and thus, better performance persistence will be an indicator of a more stable memory representation [10]. In view of the previously addressed literature, we assumed this would only be the case when the task is sufficiently practiced. Therefore, we extended the practice schedule beyond those typically used in previous studies. We examined the CI effect in complex skill learning while focusing on the level of skill persistence from the end of acquisition to retention.

## Materials and Methods

### Subjects

Forty young, healthy, right-handed subjects (mean age  = 19.6±1.3 years; range 18–23 years) took part in the experiment. All subjects were right-handed as determined by the Oldfield Handedness scale [Oldfield, 1971] (mean laterality 85.8±13.6). Subjects were randomly assigned to either of the two groups: blocked practice group (n = 20; 10 female; mean age  = 19.4±1 year; mean laterality 85.6±14.7) and randomized practice group (n = 20; 10 female; mean age  = 19.8±1.7 year; mean laterality 86.1±12.6) and were blind to the purpose of the experiment. There were no between-group differences with respect to age [*p* = 0.357], laterality quotient [*p* = 0.924] and gender was equally distributed across groups. Prior to testing, written informed consent was obtained from each subject. The protocol was approved by the local ethical committee of the University of Leuven (KU Leuven), Belgium, and was in accordance with the Declaration of Helsinki (1964).

### Instrumentation and task description

**Figure 1:**
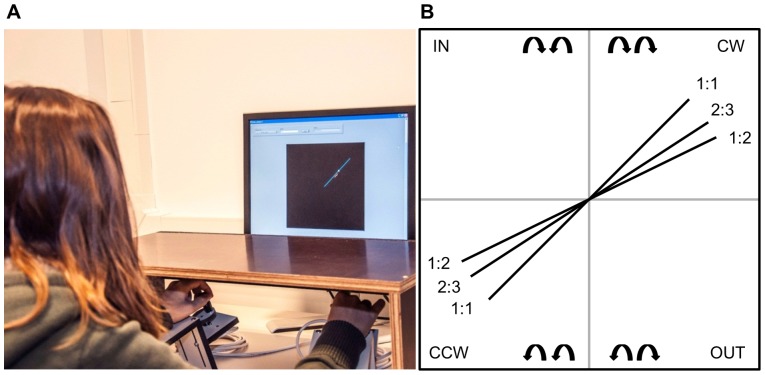
Bimanual tracking task (BTT). *(A) Task set-up*. Subjects were seated in front of a computer screen on which the task was displayed. The response apparatus consisted of two dials which were fixated on a ramp. Direct vision of the forearms was prevented by a horizontal table-top bench. ***(B) Frequency ratios and coordination directions***. Schematic drawing of the target lines shown on the screen, from which subjects can deduct the three frequency ratios (1∶1, 2∶3 and 1∶2) and coordination directions [clockwise (CW) and counterclockwise (CCW)]. The coordination directions inwards (IN) and outwards (OUT) are shown here, but are not a part of the training protocol.

A PC-based visuo-motor bimanual tracking task was used. Subjects were seated in front of a computer screen with both lower arms resting on two custom-made adjustable ramps (Figure 1A). The ramps were covered with foam to assure maximal comfort and to minimize fatigue. A dial, consisting of a flat disc (diameter 5 cm) with a vertical peg, was attached at the end of each ramp. The aim of the task was to follow a white target dot along a blue target line on the screen. To perform the required movement, subjects rotated both dials simultaneously by holding each peg between the thumb and index finger. Direct vision of hands and forearms was prevented by placing a horizontal table-top bench over the forearms of the subject. High precision shaft encoders were aligned with the axis of rotation of the dials to record angular displacement (Avago Technologies, sampling frequency  = 100 Hz, accuracy  = 0.089°). A red cursor showed the current position so that the deviation from the target dot could be corrected. The left dial controlled the vertical component of the red cursor, such that when turning it clockwise, the cursor moved up and when turning it counterclockwise, the cursor moved down. The right dial controlled the horizontal component of the red cursor, such that when turning the dial clockwise or counterclockwise, the cursor moved right and left, respectively. The gain was set to 10 units per rotation, so that 16 complete rotations of both hands were required to complete the target line that consisted of 160 arbitrary units.

A blue target line indicated the main coordination directions: both hands could rotate both clockwise (CW), both counterclockwise (CCW), inwards (IN) and outwards (OUT) (Figure 1B). The latter two coordination directions were not used in the current training protocol; however, they were used for instruction prior to testing in order to maximize understanding of the rules of the task (see below). Each coordination direction could be performed at different frequency ratios, which was visualized by the slope of the target line (Figure 1B). A target line with a 45° slope indicated a 1∶1 frequency ratio, whereby both hands were required to rotate at equal speeds. We used the convention of referring to the left hand first and the right hand second, i.e. L:R. For example, a 1∶2 frequency ratio required the right hand to move twice as fast as the left hand.

**Figure 2:**
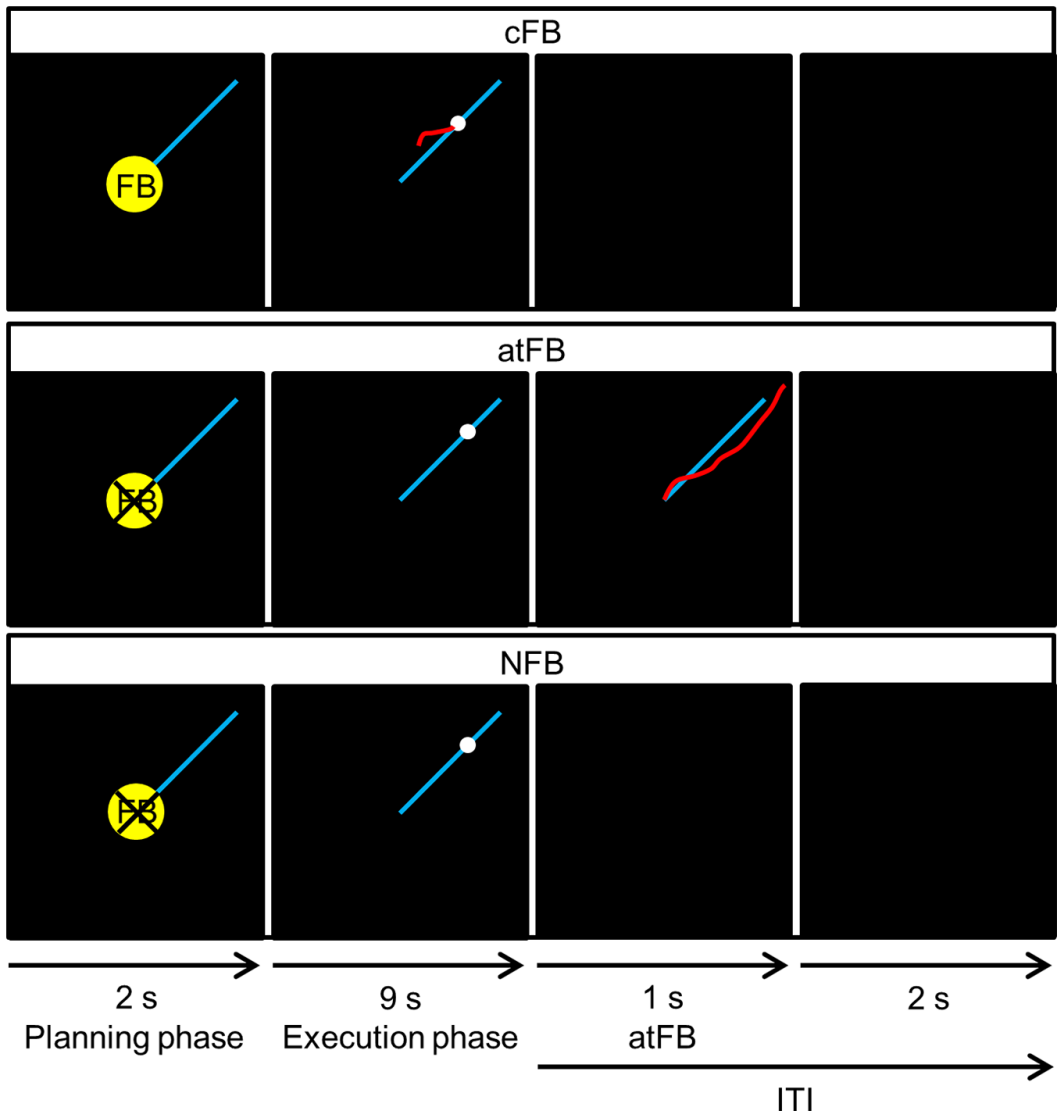
Three types of FB conditions. Concurrent visual feedback, provided by a red cursor indicative of subjects' current position, was only provided in the concurrent visual feedback (cFB) condition. In the after-trial feedback (atFB) condition, a motionless representation of the produced red line was provided after the execution phase while no feedback was provided during the execution phase. In the no feedback (NFB) condition, no concurrent or after-trial feedback was provided. Every trial started with a planning phase of 2 s where a yellow cue, which indicated whether cFB would be given in the upcoming trial, was presented. During the execution phase, the white target dot moved with constant speed along the blue target line for 9 s. In each condition, the inter-trial interval (ITI), i.e. the time between each trial where no movement was required, lasted 3 s. During ITI, atFB was provided for 1 s in the atFB condition. Instead, a black screen was presented in the cFB and NFB condition.

Three types of feedback conditions were used: concurrent visual feedback (cFB), after-trial feedback (atFB) and no feedback (NFB) (Figure 2). In all conditions, the blue target line and the white target dot were presented. In each trial, the white target dot was first covered by a yellow cue which indicated whether cFB would be given in the upcoming trial. The cue and target dot remained motionless in the center of the screen for 2 s. No movement was required, but the subject was instructed to plan the movement (planning phase). Then, an auditory cue was provided to indicate the start of the execution phase. The execution phase lasted 9 s. During the execution phase, the white target dot moved with constant speed starting from the center of the display, along the blue target line, towards the periphery. The goal of each trial was to generate the correct direction and speed by turning the dials in order to stay as close as possible to the white target dot. The inter-trial interval (ITI) lasted 3 s in which a black screen was presented. In the cFB condition, current performance was visualized online by a red cursor which contained the most recent information of the subjects' movement track (1 s), upon which movements could be corrected. During atFB trials, no red cursor was shown, but the same blue target line and the white target dot were presented. After-trial FB was provided after each trial by presenting a motionless representation of 1 s consisting of the produced red line, representing the produced movement next to the required target line, indicating the discrepancy between the produced and the required movement. In NFB trials, the blue target line and the moving white target dot were also presented, but neither cFB nor atFB was provided. Thus, in both the atFB and NFB conditions, subjects were required to track the target pathway of the frequency ratio without the guidance of concurrent visual FB.

### Study design

**Figure 3:**
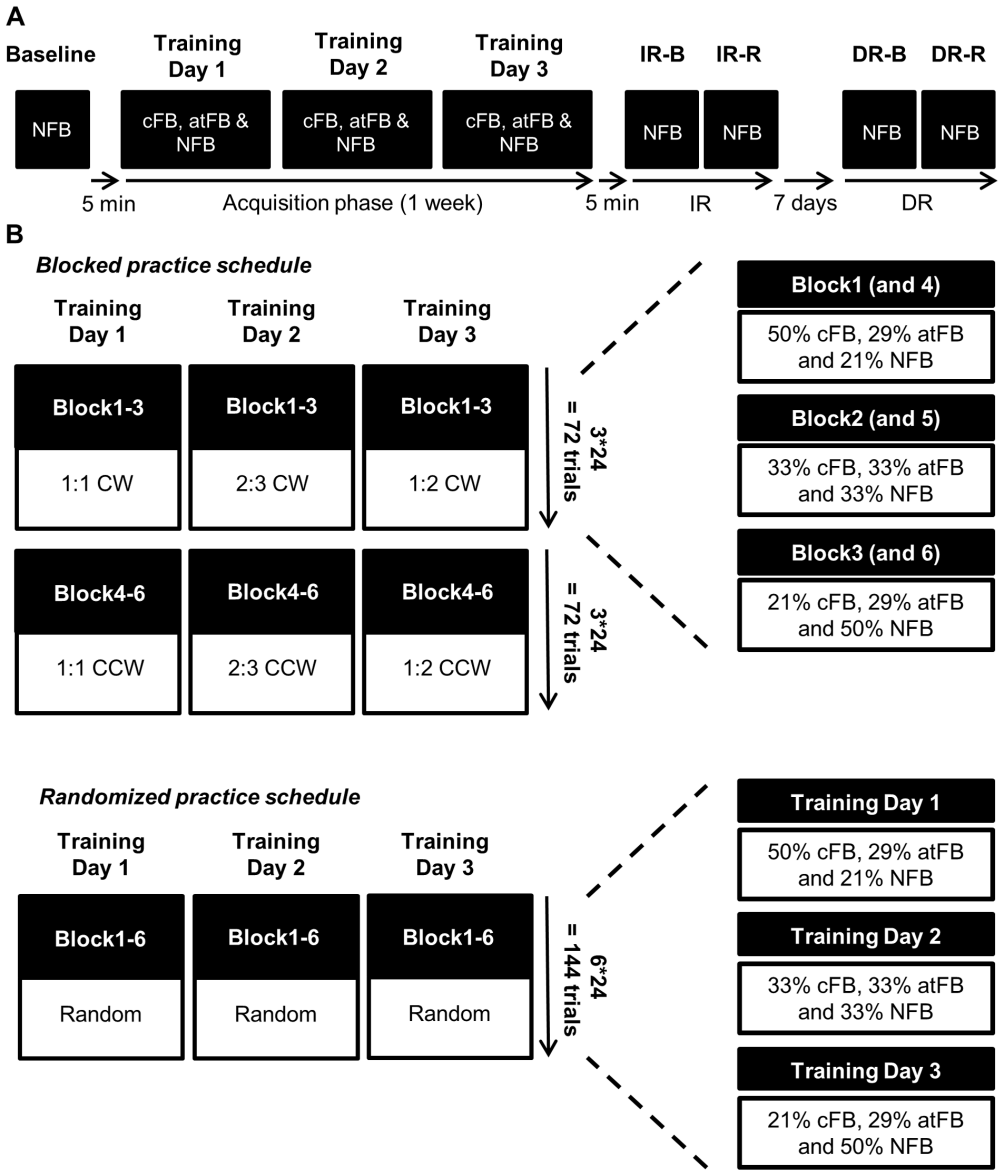
Training schedule. *(A) Training protocol*. Baseline performance was assessed without concurrent FB (NFB) on day 1 prior to training. The acquisition phase consisted of 3 training days within one week. Because of the fading feedback schedule, all 3 feedback conditions were present during each day of training. Immediate retention (IR) was conducted 5 min after the end of training day 3 and delayed retention (DR) was conducted 7 days later. Both IR and DR consisted of 2 types of retention schedule, i.e. a blocked (IR-B and DR-B) and a random (IR-R and DR-R) schedule. ***(B) Blocked and randomized practice schedule***. Subjects in the blocked practice group were exposed to one frequency ratio in both clockwise (CW) (blocks 1–3) and counterclockwise (CCW) (blocks 4–6) directions per day. In contrast, subjects in the randomized practice group were exposed to all 6 trial types (which were randomly presented) during each block, i.e. 4 trials per trial type in each block during training. The number of different feedback (cFB, atFB and NFB) trials and the degree of fading feedback within each trial type was identical in both groups. Therefore, concurrent feedback (cFB) in the blocked practice group faded over blocks 1 to 3 after which the fading feedback schedule repeated itself during the next 3 blocks. In contrast, in the randomized practice group, fading feedback was distributed over days within each trial type.

Subjects had to learn 3 different frequency ratios (1∶1, 2∶3 and 1∶2) in 2 coordination directions (CW, CCW), i.e. 6 different trial types, over 3 practice days within one week. The 6 trial types were trained either under a blocked (i.e. low CI) or randomized (i.e. high CI) practice schedule, depending on practice group. Prior to testing, subjects were informed about the basic requirements to perform the task, i.e. knowledge of the different directions and their associated rotations (CW, CCW, IN and OUT). No information was given on how to produce the different frequency ratios. To assess whether every subject understood the basic requirements of the task, a familiarization block consisting of 4 trials, i.e. a 1∶1 frequency ratio in each coordination direction (CW, CCW, IN and OUT), was conducted. For an overview of the training protocol, see Figure 3A.

#### Feedback schedule

According to the challenge point framework described by Guadagnoli and Lee [25], providing practice conditions that facilitate performance, for example by presenting high amounts of feedback until the movement representation is relatively stable, will enhance skill learning when a task is more complex. In addition, they state that the processing system is too inefficient at the early stage of learning [26], and that the learner might not be capable of interpreting information efficiently, leading to more frequent information being needed in order to yield learning [25]. In contrast, with practice (later stage of learning), the ability to process information increases, leading to better prediction to reach the goal [25], [27], [28]. Accordingly, high amounts of feedback might then be redundant for the learner [25], [27]–[29]. Therefore, to prevent reliance on feedback and to optimize learning, we made use of a fading feedback schedule [30], [31]. That is, for each trial type, we gradually reduced the number of trials in which we provided concurrent visual feedback. There were 72 trials per trial type (i.e. per frequency ratio in either CW or CCW coordination directions), across training for both practice groups. Each practice day consisted of 144 trials with six blocks of 24 trials each (Figure 3B). The numbers of cFB trials were gradually reduced (50% for trial 1–24; 33% for trial 25–48 and 21% for trial 49–72) while the NFB trials gradually increased (21%; 33% and 50% respectively) for each trial type (Figure 3B). The number of atFB trials was kept relatively constant for each trial type throughout training (29% for trial 1–24, 33% for trial 25–48 and 29% for trial 49–72). In the blocked practice group, feedback faded from block 1 to 3 after which the fading feedback schedule repeated itself for the next trial types, i.e. during every following 3 practice blocks. In the randomized practice group, all 6 trial types were randomly presented during every block across training. That is, during every training day, all 6 trial types were practiced, i.e. each trial type was presented 4 times during each block, and the trial number of each trial type (i.e. 72) was spread across the three training days. The fading feedback schedule in the randomized practice group was therefore spread over training days for each trial type. Concurrent FB was given for 50% of trials on day 1, 33% on day 2, and 21% on day 3 for all trial types. Within each training day, cFB also generally faded, starting with more cFB trials at the beginning of the training day and ending with more NFB trials. The number of cFB, atFB and NFB trials and the degree of fading within each trial type was identical in both groups. In order to see how performance without visual guidance evolved in both practice groups, i.e. where the subjects had to produce movements primarily based on an internal representation of the movement pattern instead of having the opportunity to make online corrections based on external visual information, only trials without concurrent visual feedback (65% of 432 trials), i.e. atFB and NFB, were used for analyses of acquisition phase data. For baseline and retention tests, only NFB trials were presented to subjects in order to prevent learning during these tests from online visual feedback or after trial feedback.

#### Baseline

To assess baseline performance, i.e. without prior exercise of the to be trained trial types, subjects had to perform 12 NFB trials, i.e. 2 trials per trial type, in the following blocked order: 1∶1 CW –1∶1 CCW –2∶3 CW –2∶3 CCW –1∶2 CW –1∶2 CCW.

#### Acquisition phase

The acquisition phase took 3 training days within one week. Subjects in the blocked practice group learned one frequency ratio per day in the following order: 1∶1 on day 1, 2∶3 on day 2 and 1∶2 on day 3. Each frequency ratio was learned in the CW (blocks 1–3) and in the CCW (blocks 4–6) coordination direction. Subjects in the randomized practice group were exposed to all 6 trial types in a randomized order during every block, i.e. 4 trials per trial type in each block, of each practice day. The number of practice trials for every trial type was equal for both groups. At the end of practice, a total of 432 trials were completed, of which 150 cFB trials and 282 trials without concurrent FB (132 trials atFB and 150 trials NFB). For each trial type, a total of 72 trials were practiced with 25 trials with and 47 trials without concurrent FB (22 trials atFB and 25 trials NFB). Approximately 45 minutes were needed to finish 6 practice blocks. For an overview of the acquisition phase, see figure 3B.

#### Immediate retention (IR)

Following the acquisition phase at the last day of practice (after a 5 min break), subjects were involved in an immediate retention (IR) test to assess the practiced frequency ratios. Retention accuracy is not only dependent on practice context, but also on the context in which retention is measured, i.e. a blocked or random retention test [2], [22]. Therefore, 2 retention schedules were used: a blocked IR (IR-B) and a randomized IR (IR-R). Both the IR-B and IR-R consisted of 24 NFB trials, i.e. 4 trials per trial type. During IR-B, the coordination patterns were tested following the same order as during the baseline test. After IR-B (following 1 min of rest), IR-R was conducted in which all learned coordination patterns were presented randomly. IR-B was always tested before IR-R in order to avoid confounding effects for the blocked practice group on a blocked schedule after having contact with a randomized schedule. Both IR-B and IR-R took 6 minutes to complete.

#### Delayed retention (DR)

A delayed retention (DR) test, which also consisted of a blocked DR (DR-B) and a randomized DR (DR-R), was conducted 7 days after the last day of practice. The two DR tests were exactly the same as the IR tests.

### Dependent measures

Data were recorded and analyzed with Labview (8.5) software (National Instruments, Austin, Texas, USA). The x- and y positions of the target dot and the subjects' cursor were sampled at 100 Hz. Offline analysis was carried out using Matlab R2011b and Microsoft Excel 2010. Accuracy was measured by calculating the error rate based on the average target deviation (ATD). For each trial, the target error was measured as the Euclidian distance between the target dot and the cursor position at each point in time and then averaged. Better performance is thus reflected by lower values of ATD. Because our primary focus was on the different frequency ratios and because CW and CCW movements were mainly used to provide an extra dimension of complexity to the task (as subjects needed to alternate between them), we collapsed CW and CCW data within each frequency ratio. Besides, previous research of our lab showed that the difference between CW and CCW coordination directions is negligible [32]. For the acquisition phase analyses, frequency ratio data was averaged across every set of 3 data points in time. This resulted in 16 acquisition phase data points for each frequency ratio (TR1, TR2, …, TR16). As such, TR1 for the 1∶1 frequency ratio consisted of 6 trials (3 CW and 3 CCW) without concurrent FB. Outlier trials (z>2) were discarded (6% in total of which 7% in the blocked practice group and 5% in the random practice group) from the analyses. To reduce the positive skew that was present in our data, data were log-transformed (base 10 logarithm).

### Statistical analysis

#### Frequency ratio

In order to explore whether time courses of different frequency ratios differed among each other, a full model analysis on all time points was conducted using a 2×21×3 Group (blocked, random) × Time (Baseline, TR1-16, IR-B, IR-R, DR-B and DR-R) × Frequency ratio (1∶1, 2∶3 and 1∶2) repeated measures ANOVA with Group as between-subject factor and Time and Frequency ratio as within-subject factors. First, a Time × Frequency ratio interaction effect [*F*(40,1520)  = 2.301, *p*<0.001] was found, indicating that performance over time was different for each frequency ratio. In addition, a Group × Time × Frequency ratio interaction effect [*F*(40,1520)  = 1.453, *p* = 0.034] was found indicating that performance over time significantly differed for each frequency ratio per group. This pointed towards a clear difference in time course between the 3 frequency ratios. As such, to investigate between-group differences during the acquisition phase, IR and DR, we decided to conduct separate analyses for these 3 frequency ratios.

#### Baseline

In order to assess whether there were group differences prior to practice, baseline performance was analyzed using a 2×3 Group (blocked, random) × Frequency ratio (1∶1, 2∶3 and 1∶2) repeated measures ANOVA.

#### Acquisition phase

Acquisition phase data were analyzed using a 2×16 Group (blocked, random) × Time (TR1-16) repeated measures ANOVA. To assess whether learning occurred from baseline to the end of acquisition in both practice groups, a control analysis, a 2×17×3 Group (blocked, random) × Time (Baseline, TR1-16) × Frequency ratio (1∶1, 2∶3 and 1∶2) repeated measures ANOVA, was conducted.

#### Immediate and delayed retention

To test whether performance during retention tests differed per practice group, IR and DR were analyzed using a 2×2 Group (blocked, random) × Retention schedule (blocked, random) repeated measures ANOVA.

#### Planned comparisons

Finally, we aimed to test whether the random practice group will show more performance persistence during the retention intervals than the blocked practice group. Planned a priori comparisons of least square means were conducted on the full model (2×21×3 ANOVA) to test the hypothesized differential change in performance, i.e. difference in post-acquisition processes between both groups. The two final time points of training, i.e. the end of acquisition (EoA: TR15-TR16), were taken and compared with IR and DR for both practice groups to test the interaction of Group × Time. In order to conduct these partial interaction contrasts, weights were assigned as follows. To assess the factor Group, each practice group was assigned a weight, i.e. 1 for the blocked practice group and -1 for the random practice group. For the repeated measures factor Frequency ratio, contrasts were conducted for each frequency ratio separately leading to a weight of 1 for one frequency ratio and weights of 0 for the remaining two. For the repeated measures factor Time, TR15 and TR16 both received a weight of -1, i.e. EoA, and IR-B (or DR-B for DR) and IR-R (or DR-R for DR) both received a weight of 1 in order to combine these means.

Statistical analyses were conducted using STATISTICA. For all analyses, the probability level was set at *p*<0.05, 2-sided. When significant effects were found, post hoc analyses were conducted using Tukey HSD.

## Results

Performance was compared between the randomized and blocked practice group to test CI in bimanual coordination. Performance differences were tested before practice (baseline), over the course of practice (acquisition phase), and at retention. In addition, planned comparisons were conducted in order to get a view into the efficiency of post-acquisition processes in both practice groups. As the three frequency ratios clearly differed from each other and showed an interaction effect with Group and Time (see methods), they were analyzed and illustrated separately.

### Baseline

The 2×3 Group × Frequency ratio repeated measures ANOVA did not reveal a significant main effect of Group [*F*(1,38)  = 1.857, *p* = 0.181], indicating that the performance level at baseline of both groups was comparable (blocked practice group: 1.18±0.19; random practice group: 1.12±0.14). A main effect of Frequency ratio [*F*(2,76)  = 28.294, *p*<0.001] was found. Post hoc analyses revealed that the 1∶1 frequency ratio was easier to perform than the 2∶3 frequency ratio (*p*<0.001) and 1∶2 was the most difficult frequency ratio prior to practice (*p*<0.001 and *p* = 0.007 compared with the 1∶1 and 2∶3 frequency ratio, respectively), which is in line with previous studies in our research group (e.g. Sisti et al. [32]). There was no Group × Frequency ratio interaction [*F*(2,76)  = 0.558, *p* = 0.575], suggesting that the effect of Frequency ratio was similar for both groups at baseline.

### Acquisition phase

**Figure 4:**
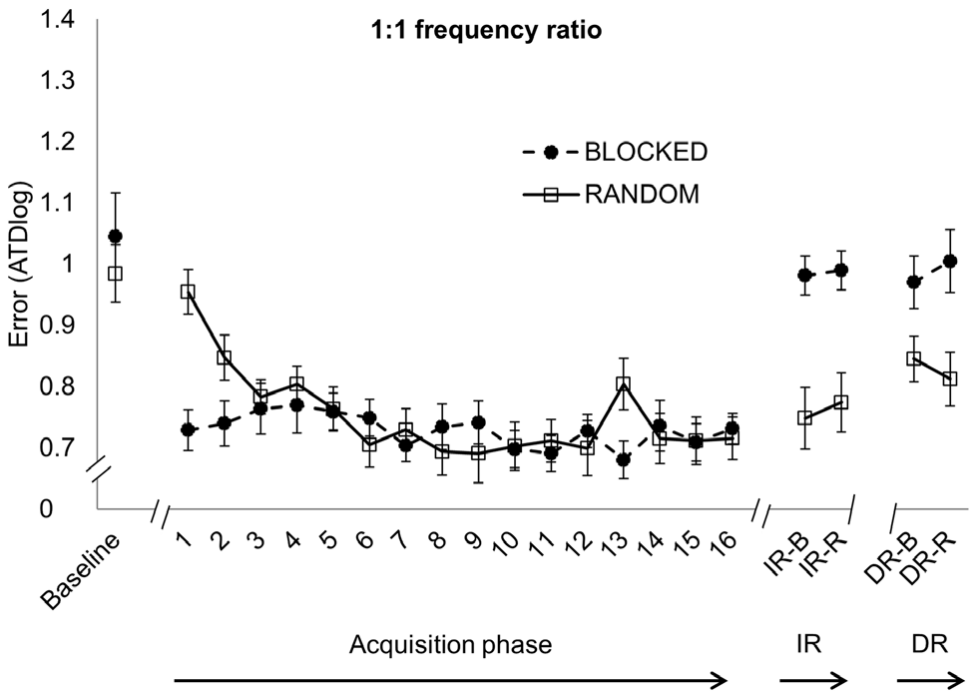
1∶1 frequency ratio. Error score (ATDlog, i.e. the log-transformed average target deviation) for baseline, acquisition phase (TR1-16), immediate retention (IR) and delayed retention (DR) (mean ± standard error) learned under either a blocked (black circles) or randomized (white squares) practice schedule. Better performance is indicated with lower levels of ATDlog.

**Figure 5:**
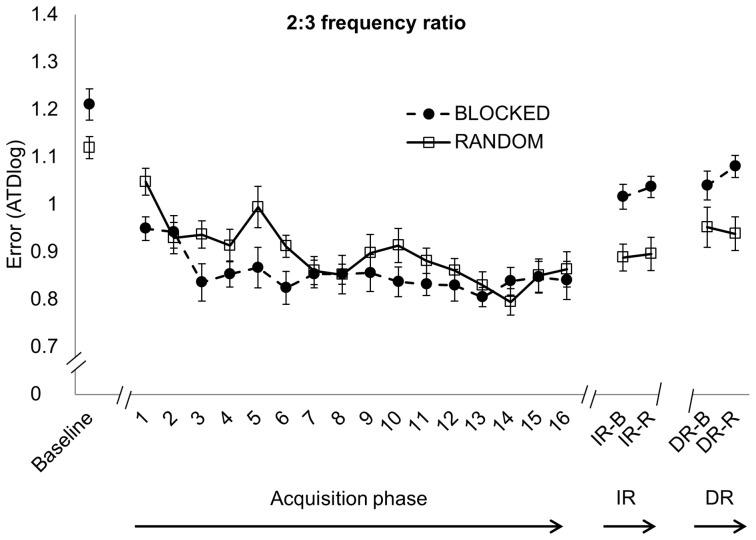
2∶3 frequency ratio. Error score (ATDlog, i.e. the log-transformed average target deviation) for baseline, acquisition phase (TR1-16), immediate retention (IR) and delayed retention (DR) (mean ± standard error) learned under either a blocked (black circles) or randomized (white squares) practice schedule. Better performance is indicated with lower levels of ATDlog.

**Figure 6:**
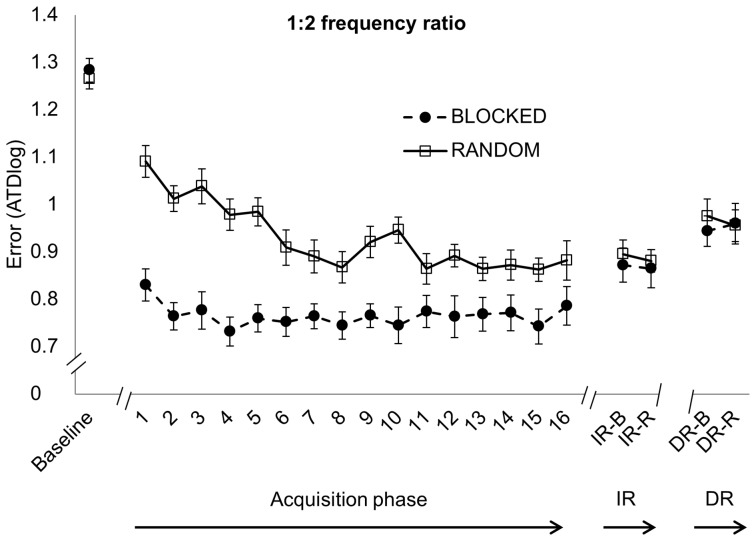
1∶2 frequency ratio. Error score (ATDlog, i.e. the log-transformed average target deviation) for baseline, acquisition phase (TR1-16), immediate retention (IR) and delayed retention (DR) (mean ± standard error) learned under either a blocked (black circles) or randomized (white squares) practice schedule. Better performance is indicated with lower levels of ATDlog.

For each frequency ratio, the 2×16 Group × Time ANOVA revealed a significant main effect of Time [*F*(15,570)  = 3.935, *p*<0.001; *F*(15,570)  = 6.342, *p*<0.001 and *F*(15,570)  = 5.811, *p*<0.001 for the 1∶1, 2∶3 and 1∶2 frequency ratio, respectively]. The 2×17×3 Group (blocked, random) × Time (Baseline, TR1-16) × Frequency ratio (1∶1, 2∶3 and 1∶2) control analysis revealed a significant main effect of Time [*F*(16,608)  = 57.183, *p*<0.001]. Post hoc analyses indicated performance improvements for each practice group from baseline to TR16 (*p*<0.001 for each frequency ratio) (Figures 4, 5 and 6). For the 1∶1 frequency ratio, there was no significant main effect of Group [*F*(1,38)  = 0.511, *p* = 0.479]. However, there was a significant Time × Group interaction effect [*F*(15,570)  = 3.243, *p*<0.001]. The randomized group started out with worse performance compared to the blocked practice group but performance became nearly as good as in the blocked practice group after approximately one-third of practice (Figure 4). For the 2∶3 frequency ratio, there was no difference between groups [*F*(1,38)  = 2.139, *p* = 0.152]. A trend towards significance was found for the Time × Group interaction [*F*(15,570)  = 1.635, *p* = 0.060] in which performance of the randomized practice group showed a less stable pattern along time with higher error peaks during practice (especially on TR5 and TR10, which was the start of training day 2 en 3 respectively for the randomized practice group) compared to the blocked practice group (Figure 5). In the 1∶2 frequency ratio, the overall performance was worse for the randomized compared to the blocked practice group, as indicated by a significant main effect of Group [*F*(1,38)  = 25.564, *p*<0.001]. This group difference was more pronounced at the beginning of practice as reflected by the significant Time × Group interaction [*F*(15,570)  = 3.510, *p*<0.001], whereby the randomized group showed more improvement than the blocked group (Figure 6).

### Immediate retention

During IR, performance of the randomized practice group was significantly better when performing the 1∶1 (0.76±0.22) and 2∶3 (0.89±0.14) frequency ratio compared to the blocked practice group (0.99±0.14 and 1.03±0.11 for the 1∶1 and 2∶3 ratio, respectively), reflected by a main effect of Group [*F*(1,38)  = 20.473, *p*<0.001 and *F*(1,38)  = 13.688, *p*<0.001 for the 1∶1 and 2∶3 frequency ratio, respectively] (Figures 4 and 5, respectively). For the 1∶2 frequency ratio, no difference between groups was found [*F*(1,38)  = 0.22, *p* = 0.642] (Figure 6). There was no main effect of Retention schedule [*F*(1,38)  = 0.292, *p* = 0.592; *F*(1,38)  = 0.731, *p* = 0.398 and *F*(1,38)  = 0.296, *p* = 0.590 for the 1∶1, 2∶3 and 1∶2 frequency ratio, respectively], indicating that randomized versus blocked testing at retention did not affect performance. There was also no interaction effect of Group × Retention schedule [*F*(1,38)  = 0.077, *p* = 0.783; *F*(1,38)  = 0.156, *p* = 0.695 and *F*(1,38)  = 0.029, *p* = 0.865 for 1∶1, 2∶3 and 1∶2 frequency ratio, respectively].

### Delayed retention

Randomized practice resulted in better delayed retention performance of the 1∶1 (0.83±0.18) and 2∶3 (0.95±0.17) frequency ratios compared with the blocked practice group (0.99±0.21 and 1.06±0.12 for the 1∶1 and 2∶3 ratio respectively), reflected by the main effect of Group [*F*(1,38)  = 8.654, *p* = 0.006 and *F*(1,38)  = 7.61, *p* = 0.008 for the 1∶1 and 2∶3 frequency ratio, respectively] (Figures 4 and 5). For the 1∶2 frequency ratio, no between group difference was found [*F*(1,38)  = 0.089, *p* = 0.767] (Figure 6). No significant main effect of Retention schedule was found for any of the frequency ratios [*F*(1,38)  = 0.001, *p* = 0.981; *F*(1,38)  = 0.009, *p* = 0.924 and *F*(1,38)  = 0.313, *p* = 0.579 for 1∶1, 1∶2 and 2∶3 frequency ratio, respectively]. In addition, there was no interaction of Group × Retention schedule on any of the frequency ratios [*F*(1,38)  = 1.159, *p* = 0.288; *F*(1,38)  = 1.308; *p* = 0.260 and *F*(1,38)  = 0.636; *p* = 0.430 for 1∶1, 2∶3 and 1∶2 frequency ratio, respectively], indicating again that randomized versus blocked retention testing did not affect performance.

### Effect of group on performance persistence

Planned comparisons revealed a significant partial interaction effect from EoA to IR in all 3 frequency ratios [*F*(1,38)  = 18.929, *p*<0.001; *F*(1,38)  = 11.064, *p* = 0.002 and *F*(1,38)  = 5.419, *p* = 0.025 for 1∶1, 2∶3 and 1∶2 frequency ratio respectively], indicating a differential change in performance between both practice groups from the end of training to immediate retention. Specifically, the randomized practice group showed more skill persistence from EoA to IR in contrast to the blocked practice group (Figures 4, 5 and 6). Partial interaction effects were also found from EoA to DR in all 3 frequency ratios [*F*(1,38)  = 7.241, *p* = 0.011; *F*(1,38)  = 4.264, *p* = 0.038 and *F*(1,38)  = 4.241, *p* = 0.046 for 1∶1, 2∶3 and 1∶2 frequency ratio respectively], indicating better performance persistence, from the EoA to DR for the randomized in contrast to the blocked practice group. Post hoc analyses revealed significant performance deterioration from EoA to IR in the blocked practice group for the 1∶1 (*p*<0.001) and 2∶3 (*p* = 0.004) frequency ratios while performance remained stable in the 1∶2 frequency ratio (*p* = 0.984). For the random practice group, performance from EoA to IR remained stable (*p* = 1) for all frequency ratios. With respect to long-term retention, the blocked practice group showed significant performance deterioration from EoA to DR in all frequency ratios (*p*<0.001 for the 1∶1 and 2∶3 frequency ratios and *p* = 0.002 for the 1∶2 frequency ratio) while performance of the random practice group remained stable over this one week period (*p* = 0.889 for the 1∶1 frequency ratio and *p* = 1 for the 2∶3 and 1∶2 frequency ratio).

## Discussion

The purpose of this study was to explore the CI effect in a complex bimanual coordination task. First, we hypothesized that a random practice schedule would have detrimental effects during the acquisition phase, but would result in better immediate and delayed retention performance. There was an overall pattern in which the random practice group performed considerably worse at the beginning of the acquisition phase, but with practice, performance of the random practice group progressed towards the performance level of the blocked group. As expected, immediate and delayed retention were superior after random compared to blocked practice, although this effect was absent in the more difficult 1∶2 ratio. Finally, we hypothesized that randomized practice would show more performance persistence during retention intervals, i.e. 5 minutes and one week after the end of practice. This expectation was confirmed in all three frequency ratios.

As stated in the introduction, both the elaboration and action-plan reconstruction hypotheses suggest that enhanced cognitive effort and processing during high CI is responsible for beneficial learning effects. However, none of these hypotheses entail different predictions regarding CI in relation with complex skill learning, in which high attention and memory demands are inherent to the nature of the task itself, and thus requiring more cognitive effort in contrast to simple tasks. We will discuss the effects of CI during complex bimanual coordination task learning with a focus on the temporal evolution of memory processes.

### Acquisition phase

One key feature of the CI effect is that a blocked practice schedule facilitates performance during the acquisition phase compared to a random practice schedule [4], [33], [34]. Although our results did not show a main effect between groups, which may be due to the longer training schedule, there was a robust interaction in which performance of the randomized group progressed towards the blocked group in each of the frequency ratio conditions. In line with other studies, better performance of the blocked group was more pronounced early in practice [2], [22]. As confirmed by previous work, longer practice schedules can even result in the random practice group to outperform the blocked group during training [17]. This suggests that the detrimental effects of random practice during the acquisition phase can be overcome. Due to the presumed higher difficulty level of the 1∶2 frequency ratio, the CI effect could not be overcome during practice. This may imply that it takes more time for the random practice group to stabilize and reach a similar performance level as the blocked group when the difficulty level increases. Some additional points regarding the performance changes in the blocked practice group across the acquisition phase are worth noting. First, performance differences between the first acquisition blocks and initial baseline performance in the blocked practice group are more prominent than appears at first sight. Moreover, performance improvements from baseline to TR16 in the blocked practice group were present. Furthermore, each data point in the acquisition phase (e.g. TR1) already consisted of 6 trials (3 CW and 3 CCW). In addition, only trials without concurrent FB are shown in the figures. Because of the fading feedback schedule, more concurrent FB trials were offered at the beginning of acquisition. As stated above, better performance of the blocked group was clearly more pronounced early in practice [2], [22]. Maslovat et al. [17], for example, tested the CI effect while learning a bimanual coordination task by using an extensive practice schedule (100 trials per coordination pattern). Regarding the acquisition data, the authors showed that the blocked practice group did not improve their performance significantly on the 90° coordination pattern and, little performance improvements were present after the first 18 acquisition trials on the 45° coordination pattern. In line with these interpretations, ceiling effects were indeed reached very early in the blocked practice group.

### Retention

Retention performance was not dependent on the context in which retention was obtained (i.e. blocked or random retention schedule). In this respect, the specificity of learning hypothesis [35], which predicts that conditions during practice which most closely match the criterion conditions will be most effective for learning that criterion, is not supported here. This is consistent with the study of Shea et al. [22] in which the context of retention had no influence on performance following a random practice schedule. However, in contrast to Shea et al. [22], retention schedule performance was also not influenced following a blocked practice schedule. Reviews have reported mixed results regarding the magnitude of performance benefit following a random practice schedule [4], [33]. In the current study, performance differences during retention favoring the random group were confirmed in the 1∶1 and 2∶3 frequency ratios, while no performance differences were observed in retention tests of the 1∶2 frequency ratio. Brady [33] noted that the effect of CI could be a function of the difficulty of a task. In line with this notion, advantages of high CI were found when learning a drawing task; however, the advantage tended to be more pronounced in the simplest versions of the task in contrast to the most difficult version, even though this effect was not significant [20]. Wulf and Shea [5] suggested that there might be a link between the amount of practice and task difficulty. They stated that random practice is more effective when practitioners become more experienced in a complex task, such that the cognitive demands, needed to complete the tasks, are reduced. In line with this notion, random practice in complex tasks may lead to a system overload early in practice, when attention, memory and motor demands are high [5]. The higher memory or motor demands required to perform the 1∶2 ratio, in combination with high CI during practice, may have overloaded the system and, in turn, disrupted the beneficial effects of high CI in this ratio. The CI effect in the 1∶2 frequency ratio may thus be increased by increasing the amount of practice.

### Performance persistence

We hypothesized that the random as compared to the blocked practice group would show more performance persistence during the transition from the acquisition to the retention phase. This was confirmed in all three frequency ratios. Moreover, while performance of the random practice group remained stable over a one week period (EoA to DR), performance of the blocked practice group showed a significant deterioration during this time interval. Kantak and Winstein [10] mentioned the importance of the time interval between the end of acquisition and retention tests in order to reveal performance changes, which provides insight into distinct memory processes. A key question here is whether better performance persistence in the random group was a result of more efficient post-acquisition consolidation processes. At first sight, the answer seems to be positive as consolidation is defined as a set of post-acquisition changes wherein a new skill is strengthened [36]. However, these results should be interpreted with caution, as the three distinct memory processes (encoding, consolidation and retrieval) are interrelated and may partially overlap in the temporal domain [10]. Especially in this study, where practice is divided over multiple days, these processes are even more intertwined.

First, it is known that motor performance measured during practice can be related to two main effects of practice: relatively permanent effects, which are conceptualized as learning effects, and temporary or transient effects, which often vanish when the manipulation is removed [37]. Both the elaboration and the action-plan reconstruction hypotheses assume that the greater investment in task-related cognitive processing during random practice will result in the development of a stronger memory representation, which leads to more permanent effects [34]. By contrast, blocked practice will provoke more temporary or transient effects, which are beneficial for acquisition performance. Thus, the faster decrease in performance following the acquisition phase in the blocked group might be explained by (a) a more fragile memory representation as a result of different encoding processes than the random group, (b) the fading of the beneficial temporary effects that are present during practice, or a combination of both explanations. Second, as practice was distributed over three practice days (within a one week period), subjects had the potential to consolidate, and thus strengthen the memory representation in-between practice sessions. As the blocked group practiced only one frequency ratio a day, it was not possible to assess between-practice consolidation processes, which may be a limitation of the present study.

As already stated in the introduction; studies examining the CI effect in complex motor skills have led to contrary results [5]. Tsutsui et al. [21] examined the effect of CI in learning new patterns of bimanual coordination using multiple days of training. The authors did not find any effect of CI when all coordination patterns were practiced within each practice day. However, when the blocked practice group learned each pattern on separate days, typical CI effects were reported. Albaret and Thon [20] examined the effects of task complexity on CI using a unimanual drawing task. The authors demonstrated retention benefits following random practice; however, the advantage tended to be more pronounced in the simplest versions of the task in contrast to the most difficult. By contrast, the authors did not find any influence of CI when accuracy of orientation, i.e. directional error, was taken into account. Nevertheless, in both papers [20], [21], performance stability from end of acquisition to delayed retention was not statistically compared between groups.

Finally, it is important to consider a possible confound in our design regarding different retention delays in the blocked practice group as a result of a fixed practice order. Please note that our experiment was designed this way because we hoped for the best possible learning effects by providing incremental task difficulty. One might argue that the decrement in performance from EoA to IR in the blocked practice group might be mediated by different retention intervals and thus reflects differential forgetting, i.e. more skill deterioration in the 1∶1 frequency ratio (minimum delay of 2 days from EoA to IR) compared with the 1∶2 frequency ratio (5 minute delay from EoA to IR). Therefore, a control experiment (n = 25) was conducted in which frequency ratios were presented in a blocked manner, but counterbalanced over practice days. Subjects were randomly assigned to one of 6 different practice orders (for more details, see File S1). Results indicated that the behavioral pattern of the counterbalanced blocked practice group was similar to the fixed blocked practice group described in the current paper. This provides compelling evidence that the effects in the current experiment are not due to different retention intervals.

In summary, we can conclude that random practice resulted in better skill persistence in a complex bimanual coordination task. Specifically, while the blocked practice group showed significant skill deterioration over a one week period, performance of the random practice group remained stable. This effect was evident in all three coordination patterns of various difficulty levels. Although better skill persistence following a random practice schedule was found, the random group could not outperform the blocked group in the most difficult frequency ratio.

The finding that high as compared to low CI led to better skill persistence in a complex bimanual coordination task, even one week after the practice period, is important for future research. Following Kantak and Winstein [10], we agree that examining the temporal evolution of performance will provide more insight into the mechanisms that implement the learning-performance distinction. In the past, research regarding the CI effect in complex tasks yielded mixed results [5]. However, if we want to provide adequate recommendations for practical settings, we have to examine to what extent the CI effect is generalizable to complex skill learning.

## Supporting Information

Figure S1 **Overall behavioral pattern.** Effect of practice order (fixed versus counterbalanced blocked practice) on error score (ATDlog, i.e. the log-transformed average target deviation) for baseline, acquisition phase (TR1-16), immediate retention (IR) and delayed retention (DR) (mean ± standard error). Better performance is indicated with lower levels of ATDlog.
(TIF)

Figure S2 **1:1 frequency ratio.** Effect of practice order (fixed versus counterbalanced blocked practice) on error score (ATDlog, i.e. the log-transformed average target deviation) for baseline, acquisition phase (TR1-16), immediate retention (IR) and delayed retention (DR) (mean ± standard error). Better performance is indicated with lower levels of ATDlog.
(TIF)

Figure S3 **2:3 frequency ratio.** Effect of practice order (fixed versus counterbalanced blocked practice) on error score (ATDlog, i.e. the log-transformed average target deviation) for baseline, acquisition phase (TR1-16), immediate retention (IR) and delayed retention (DR) (mean ± standard error). Better performance is indicated with lower levels of ATDlog.
(TIF)

Figure S4 **1:2 frequency ratio.** Effect of practice order (fixed versus counterbalanced blocked practice) on error score (ATDlog, i.e. the log-transformed average target deviation) for baseline, acquisition phase (TR1-16), immediate retention (IR) and delayed retention (DR) (mean ± standard error). Better performance is indicated with lower levels of ATDlog.
(TIF)

File S1 **Fixed versus counterbalanced blocked practice.** In order to test whether the different retention delays in the blocked practice group influenced our results, a control experiment was conducted.
(DOCX)

## References

[1] Battig WF (1979) The flexibility of human memory. In: Cermak LS, Craik FIM, editors. Levels of processing in human memory. Hillsdale, NJ: Erlbaum. 23–44.

[2] Shea JB, Morgan R (1979) Contextual interference effects on the acquisition, retention, and transfer of a motor skill. J Exp Psychol Hum Learn Mem 5: 179–187.

[3] Magill RA (2011) Motor learning and control: Concepts and applications. New York: McGraw-Hill.

[4] Magill RA, Hall KG (1990) A review of the contextual interference effect in motor skill acquisition. Hum Mov Sci 9: 241–289.

[5] Wulf G, Shea CH (2002) Principles derived from the study of simple skills do not generalize to complex skill learning. Psychon Bull Rev 9: 185–211. 12120783 10.3758/bf03196276

[6] Shea JB, Zimny ST (1983) Context effects in memory and learning movement information. In: Magill RA, editor. Memory and control of action. Amsterdam: North-Holland 345–366.

[7] Lee TD, Magill RA (1983) The locus of contextual interference in motor-skill acquisition. J Exp Psychol Learn Mem Cogn 9: 730–746.

[8] Lee TD, Magill RA (1985) Can forgetting facilitate skill acquisition? In: Goodman D, Wilberg RB, Franks IM, editors. Differing perspectives in motor learning, memory, and control. Amsterdam: Elsevier. 3–22.

[9] Young DE, Cohen MJ, Husak WS (1993) Contextual interference and motor skill acquisition: On the processes that influence retention. Hum Mov Sci 12: 577–600.

[10] Kantak SS, Winstein CJ (2012) Learning-performance distinction and memory processes for motor skills: A focused review and perspective. Behav Brain Res 228: 219–231. 22142953 10.1016/j.bbr.2011.11.028

[11] Lin CH, Fisher BE, Winstein CJ, Wu AD, Gordon J (2008) Contextual interference effect: elaborative processing or forgetting-reconstruction? A post hoc analysis of transcranial magnetic stimulation-induced effects on motor learning. J Mot Behav 40: 578–586. 18980910 10.3200/JMBR.40.6.578-586

[12] Lin CH, Winstein CJ, Fisher BE, Wu AD (2010) Neural correlates of the contextual interference effect in motor learning: A transcranial magnetic stimulation investigation. J Mot Behav 42: 223–232. 20570818 10.1080/00222895.2010.492720

[13] Kantak SS, Sullivan KJ, Fisher BE, Knowlton BJ, Winstein CJ (2010) Neural substrates of motor memory consolidation depend on practice structure. Nat Neurosci 13: 923–925. 20622872 10.1038/nn.2596

[14] Tanaka S, Honda M, Hanakawa T, Cohen LG (2010) Differential contribution of the supplementary motor area to stabilization of a procedural motor skill acquired through different practice schedules. Cereb Cortex 20: 2114–2121. 20038545 10.1093/cercor/bhp276 PMC2923213

[15] Smith PJ, Davies M (1995) Applying contextual interference to the Pawlata roll. J Sports Sci 13: 455–462. 8850571 10.1080/02640419508732262

[16] Wrisberg CA, Liu Z (1991) The effect of contextual variety on the practice, retention, and transfer of an applied motor skill. Res Q Exerc Sport 62: 406–412. 1780563 10.1080/02701367.1991.10607541

[17] Maslovat D, Chus R, Lee TD, Franks IM (2004) Contextual interference: single task versus multi-task learning. Motor Control 8: 213–233. 15118203 10.1123/mcj.8.2.213

[18] Hebert EP, Landin D, Solmon MA (1996) Practice schedule effects on the performance and learning of low- and high-skilled students: An applied study. Res Q Exerc Sport 67: 52–58. 8735994 10.1080/02701367.1996.10607925

[19] Jarus T, Gutman T (2001) Effects of cognitive processes and task complexity on acquisition, retention, and transfer of motor skills. Can J Occup Ther 68: 280–289. 11765667 10.1177/000841740106800504

[20] Albaret JM, Thon B (1998) Differential effects of task complexity on contextual interference in a drawing task. Acta Psychol (Amst) 100: 9–24. 9844553 10.1016/s0001-6918(98)00022-5

[21] Tsutsui S, Lee TD, Hodges NJ (1998) Contextual interference in learning new patterns of bimanual coordination. J Mot Behav 30: 151–157. 20037030 10.1080/00222899809601332

[22] Shea CH, Kohl R, Indermill C (1990) Contextual interference: contributions of practice. Acta Psychol (Amst) 73: 145–157.

[23] Boutin A, Blandin Y (2010) On the cognitive processes underlying contextual interference: Contributions of practice schedule, task similarity and amount of practice. Hum Mov Sci 29: 910–920. 20822819 10.1016/j.humov.2010.07.011

[24] Swinnen SP, Wenderoth N (2004) Two hands, one brain: Cognitive neuroscience of bimanual skill. Trends Cogn Sci 8: 18–25. 14697399 10.1016/j.tics.2003.10.017

[25] Guadagnoli MA, Lee TD (2004) Challenge point: A framework for conceptualizing the effects of various practice conditions in motor learning. J Mot Behav 36: 212–224. 15130871 10.3200/JMBR.36.2.212-224

[26] Adams JA (1971) A closed-loop theory of motor learning. J Mot Behav 3: 111–149. 15155169 10.1080/00222895.1971.10734898

[27] Marteniuk RG (1976) Information processing in motor skills. New York: Holt, Rinehart, and Winston.

[28] Kay H (1970) Analyzing motor performance. In: Connolly K, editor. Mechanisms of motor skill development. New York: Academic Press.

[29] Schmidt RA, Lee TD (1999) Motor control and learning: A behavioral emphasis: Human Kinetics.

[30] Kovacs AJ, Shea CH (2011) The learning of 90 degrees continuous relative phase with and without Lissajous feedback: External and internally generated bimanual coordination. Acta Psychol (Amst) 136: 311–320. 21216384 10.1016/j.actpsy.2010.12.004

[31] Winstein CJ, Schmidt RA (1990) Reduced frequency of knowledge of results enhances motor skill learning. J Exp Psychol Learn Mem Cogn 16: 677–691.

[32] Sisti HM, Geurts M, Clerckx R, Gooijers J, Coxon JP, et al (2011) Testing multiple coordination constraints with a novel bimanual visuomotor task. PLoS One 6: e23619. 21858185 10.1371/journal.pone.0023619 PMC3157395

[33] Brady F (1998) A theoretical and empirical review of the contextual interference effect and the learning of motor skills. Quest 50: 266–293.

[34] Lee TD, Simon D (2004) Contextual interference. In: Wiliams AM, Hodges NJ, editors. Skill acquisition in sport: research, theory and practice. London: Routledge. 29–44.

[35] Barnett ML, Ross D, Schmidt RA, Todd B (1973) Motor skills learning and the specificity of training principle. Res Q 44: 440–447. 4532277

[36] Robertson EM, Pascual-Leone A, Miall RC (2004) Current concepts in procedural consolidation. Nat Rev Neurosci 5: 576–582. 15208699 10.1038/nrn1426

[37] Schmidt RA, Bjork RA (1992) New conceptualizations of practice – Common principles in 3 paradigms suggest new concepts for training. Psychol Sci 3: 207–217.

