# Supplementary material for: Contextual Interference in Complex Bimanual Skill Learning Leads to Better Skill Persistence
Source: PLoS One. 2014 Jun 24;9(6):e100906. doi: 10.1371/journal.pone.0100906 (PMC4069194; doi:10.1371/journal.pone.0100906)
Supplement: File S1 — Fixed versus counterbalanced blocked practice. In order to test whether the different retention delays in the blocked practice group influenced our results, a control experiment was conducted. (DOCX) [file pone.0100906.s005.docx]

# SUPPORTING INFORMATION S1

# Fixed versus counterbalanced blocked practice

## Introduction

It is important to consider a possible confound in our design regarding different retention delays in the blocked practice group as a result of a fixed practice order. One might argue that the decrement in performance from the end of acquisition (EoA) to immediate retention (IR) in the blocked practice group could be mediated by different retention intervals and thus reflects differential forgetting, i.e. more skill deterioration in the 1:1 frequency ratio (minimum delay of 2 days from EoA to IR) compared with the 1:2 frequency ratio (5 minute delay from EoA to IR). Post hoc analyses led us to hypothesize that the different retention delays in the blocked practice group did not influence our data since there was no significant performance deterioration from IR to delayed retention (DR) (*p* = 1 for all frequency ratios). If our findings would have been influenced by different retention intervals, these effects would be minimized over a one week period. However, in order to be certain that this possible order effect did not take place, a control group, i.e. counterbalanced blocked practice group, was tested in which frequency ratios were counterbalanced over practice days.

## Materials and Methods

### Subjects

Twenty-five subjects (13 female; mean age = 19.6 ± 2.2 years; mean laterality 86.2 ± 19.1) took part in the control experiment, i.e. **counterbalanced blocked practice group**, and were compared with subjects of the blocked practice group described in the paper, i.e. **fixed blocked practice group**. Subjects were blind to the purpose of the experiment. There were no between group differences with respect to age [*p* = 0.226] and laterality quotient [*p* = 0.910]. Prior to testing, written informed consent was obtained from each subject. The protocol was approved by the local ethical committee of the University of Leuven (KU Leuven), Belgium, and was in accordance with the Declaration of Helsinki (1964).

### Task and study design

The instrumentation and task used was identical to the one used in the paper. The study design for the counterbalanced blocked practice group was nearly identical to the one used in the fixed blocked practice group. In the counterbalanced blocked practice group, frequency ratios were presented in a blocked manner, but counterbalanced over training days with 6 different practice orders. Subjects were randomly assigned to one of the following practice orders on day 1, 2 and 3 respectively: **1)** 1:1, 2:3 and 1:2 (5 subjects) **2)** 1:2, 2:3 and 1:1 (4 subjects) **3)** 2:3, 1:1 and 1:2 (4 subjects) **4)** 1:2, 1:1 and 2:3 (4 subjects) **5)** 2:3, 1:2 and 1:1 (4 subjects) **6)** 1:1, 1:2 and 2:3 (4 subjects). Furthermore, frequency ratios were counterbalanced in baseline, IR-B and DR-B tests. All other parameters were kept the same.

### Statistical analysis

Dependent measures were identical to the one used in the paper. To test the influence of practice order, a 2 x 21 x 3 Practice order (fixed, counterbalanced) x Time (Baseline, TR1-16, IR-B, IR-R, DR-B and DR-R) x Frequency ratio (1:1, 2:3 and 1:2) repeated measures ANOVA with Practice order as between-subject factor and Time and Frequency ratio as within-subject factors was conducted.

## Results and Discussion

No main effect of Practice order was found [*F*(1,43) = 1.662, *p* = 0.204]. In addition, there was no Practice order x Time interaction effect [*F*(20,860) = 0.637, *p* = 0.886] which indicated a similar behavioral pattern over time for both experimental groups (Figure S1). Finally, no Practice order x Time x Frequency ratio interaction effect was observed [*F*(40,1720) = 1.305, *p* = 0.097] indicating that, within each frequency ratio, the behavioral pattern over time was comparable between the fixed and counterbalanced blocked practice group (Figure S2, Figure S3 and Figure S4).

Accordingly, this control experiment provides strong evidence that the effects described in the main manuscript are not due to different retention intervals.
